# Periodical Progress in Ecophysiology and Ecology of Grassland

**DOI:** 10.3390/plants12122244

**Published:** 2023-06-08

**Authors:** Bingcheng Xu, Zhongming Wen

**Affiliations:** 1 State Key Laboratory of Soil Erosion and Dryland Farming on the Loess Platea, Northwest A&F University, Yangling 712100, China; 2 College of Grassland Agriculture, Northwest A&F University, Yangling 712100, China

As one of the most important ecosystems on the planet, grasslands serve a variety of purposes in ecology, economy, culture and entertainment. Due to overuse, insufficient input, poor maintenance and climate change, natural grasslands have become one of the most degraded ecosystems and are experiencing numerous challenges. To better understand the dynamics of biomass elaboration and the renewal of natural grassland, it is necessary to conduct a systematical analysis of ecophysiological and ecological traits of grassland communities and their key species. With this framework, “Ecophysiology and Ecology of Grassland” focuses on recent advancements concerning integrated research from species to ecosystem levels in terms of natural grasslands as well as artificial grasslands, in response to human disturbances, abiotic stresses and climate change. The following is a brief summary of the topics and applications that comprise this Special Issue.

Fang et al., (2021) [1] studied the spatial patterns and potential drivers of leaf stoichiometry and herb biomass from 15 sites spreading from south to north along a 500 km latitudinal gradient on the Loess Plateau. The findings demonstrated a strong relationship between herb biomass and leaf N and P contents and environmental driving factors, including slope, soil P content and latitude, altitude, mean annual rainfall and mean annual temperature, which can be used to inform future ecological restoration efforts and policy adjustments in the region as well as to offer basic regional data for global-scale research.

Zhang and Wang et al., (2022) [2] investigated the responses of plants, soil bacteria, and fungal diversity to climate change (especially rainfall patterns and air temperature) in the desert grassland of the Ningxia Hui Autonomous Region of China. The results indicated that increased precipitation promoted root biomass growth more than aboveground living biomass, and changing precipitation and increasing temperature, as well as their interaction, primarily altered the plant diversity, soil bacteria and fungal diversity but had no significant impact on plant biomass production, organic carbon, total nitrogen and total phosphorus contents of plants. The study sheds light on the diversity and variability of desert grassland plants and soil microorganisms in relation to climate warming and precipitation change.

Zhang and Xie et al., (2022) [3] studied the dynamics of grassland vegetation and compared the effect of drought in the Mongolian Plateau (MP) from 2000 to 2013 using a multi-index method that included coverage (Fv), surface bareness (Fb), and net primary production (NPP). Fv and NPP exhibited an increasing trend (0.18 vs. 0.43), while Fb showed a decreasing trend, with a value of −0.16. Generally, the grassland in the MP showed a tendency to recover, and the response is region-specific (positive reaction mainly distributed in the middle of MP). The results provided a scientific foundation for guiding ecological, environmental improvement and drought prevention in typical farming and pastoral areas.

Song et al., (2022) [4] investigated the effect of the water deficit on the eco-adaptation of common grassland species and conducted a water control experiment to unveil the ecophysiology of *Glycyrrhiza uralensis* in response to different water stress gradients. The species is the dominant species in the natural restoration of the desert steppe. *G. uralensis* was shown to maintain water content and turgor pressure under water stress, promote root biomass accumulation, and improve water use efficiency, indicating that it possesses a water-conservation strategy to avoid dehydration and tolerate drought. The study also investigated the biomass allocation, water use efficiency, and physiological and morphological characteristics of *G. uralensis*, which provided scientific support for adopting the species to restore the grassland.

Guo et al., (2022) [5] investigated the leaf stoichiometry of the widely distributed species *Stellera chamaejasme* from the Inner Mongolian Plateau (IM) and Qinghai–Tibet Plateau (QT) in China and evaluated its relationships with environmental variables. There was no discernible difference between leaf C, N, and P content in *S. chamaejasme* from the QT and IM, but the leaf K concentration was significantly higher in QT than that in IM, and there was no significant correlation between leaf ecological stoichiometry of *S. chamaejasme* and soil physicochemical properties. According to the findings, *S. chamaejasme* can adapt to changing environments by adjusting its relationships with climatic or soil factors to improve its survival in degraded grasslands.

Jin et al., (2022) [6] investigated the photosynthetic characteristics and leaf economic traits of three dominant species (two grass species: *Bothriochloa ischaemum* and *Stipa bungeana*; one leguminous subshrub: *Lespedeza davurica*) in a semiarid grassland community on the Loess Plateau of China. The community was continuously treated with nitrogen (N) and phosphorus (P) inputs for three years, and the study suggests that N and P addition shifted leaf economic traits towards a greater light harvesting ability and elevated photosynthesis in the three dominant species due to evident N and P synergetic effects, and this was achieved by species-specific responses in leaf functional traits. The results provide insights into grassland restoration and the assessment of community development in the context of atmospheric N deposition and intensive agricultural fertilization.

In the same experiment as Jin et al., (2022) [6], Yang et al., (2022) [7] used trait-based approaches to analyze and compare the relative contributions of plant functional traits to grassland productivity under N and/or P addition on the semiarid Loess Plateau. The results showed that the linkages between plant functional traits and the relative biomass of species differed under different N and P addition levels, and dominant species traits could predict ecosystem functioning (productivity) on the semiarid grassland. The study validated the mass ratio hypothesis and highlighted the close linkages between community-level functional traits and grassland productivity. The study advances our understanding of the mechanisms underpinning biodiversity–ecosystem functional relationships and has significant implications for semiarid grassland management.

Zuffo et al., (2022) [8] compared the tolerance of nine cultivars belonging to five species of perennial tropical forage grasses in Brazil growing in pots with controlled soil water conditions. After comparing and evaluating of twelve tolerance indices, the mean production (MP), drought resistance index (DI), stress tolerance index (STI), geometric mean production (GMP), yield index (YI), modified stress tolerance (k_2_STI) and harmonic mean (HM) were selected as the most suitable parameters for identifying forage grass cultivars with greater water stress tolerance and a high potential for shoot biomass production under severe water stress condition. The study is significant in terms of selecting metrics for assessing and identifying water-stress-tolerant genotypes.

Duan et al., (2022) [9] evaluated plant functional traits (PFTs) of 171 herb plots in 57 sites (from varied topography and herb types) using 29 variables categorized into four types from a typical Soil and Water Conservation Demonstration Park (SWDP) on the Loess Plateau of China. The study attempted to quantify the effects of topographic conditions, soil factors and vegetation structure on PFTs. The results showed that the topographic conditions and soil properties had direct effects on plant functional traits, with slope having the most weight in topographic conditions and maximum water capacity (MWC) having the most the highest weight in soil properties, followed by soil water content (SWC). The study adds to our understanding of the mechanisms of resource utilization, competition and adaptation of plants in heterogeneous habitats through quantifying the association between distinct factors. 

Mugloo et al., (2023) [10] reported the yield and nutritional contents of grass and legume species in Kashmir Valley’s rangelands. The study area included grazed, protected, and seed-sown sites. The results showed that aboveground biomass (AGB) and total biomass yield were highest in the protected sites of the central Kashmir region, whereas belowground biomass (BGB) was highest in the protected sites of the southern Kashmir region. The study indicated that moderate grazing had moderate effects on biomass production, and species in different regions responded differently to disturbance, implying site-specific and species-specific traits in response to disturbance and climate change.

In a one-year plot experiment, Ma et al., (2023) [11] simulated combinations of different species compositions (1, 2, 4, 6 species and 8 species), and monitored the phenology of alfalfa and determined the related functional traits such as light acquisition traits (plant height and relative height, leaf mass and area, leaf length and width, and specific leaf area) and nutrient acquisition traits (leaf carbon content (LCC), leaf nitrogen content (LNC), leaf C/N ratio (LCC/LNC), biomass and abundance, relative biomass and abundance) after N addition. The results showed that the effect of N addition and plant diversity on flowering phenology was driven by the intraspecific variation in functional traits, and such alteration would influence the flower numbers and plant reproductive strategy.

He et al., (2023) [12] reviewed the studies on the structure and stability of grassland ecosystems, summarized the progress and future directions, and suggested ideas for improving the ecosystem service capacity of grasslands for Karst Desertification Control (KDC). Based on the background of KDC and its geographical characteristics, they proposed three insights to optimize the spatial allocation, enhance the grassland stability for rocky desertification control and coordinate the relation between grassland structure and stability. This study provided support for grassland managers and relevant policymakers to improve the structure, stability, and service capacity of grassland ecosystems in the karst region.

As Guest Editors, we sincerely thank all the authors who contributed to our Special Issue. We appreciate the efforts of all the reviewers and academic editors in evaluating the selected manuscripts and upholding the high standards of peer review. We anticipate that the papers in this Special Issue will serve as a springboard for future work on the ecophysiology and ecology of grasslands in the face of anthropogenic activities and climate change.
